# Association of Mycobacterium Tuberculosis Lineages with IFN-γ and TNF-α Gene Polymorphisms among Pulmonary Tuberculosis Patient

**DOI:** 10.4084/MJHID.2014.015

**Published:** 2014-02-16

**Authors:** Mohammad Varahram, Parissa Farnia, Mohammad Javad Nasiri, Mona Afraei Karahrudi, Mehdi Kazempour Dizagie, Ali Akbar Velayati

**Affiliations:** Mycobacteriology Research Centre, National Research Institute of Tuberculosis and Lung Disease [NRITLD], Masih Daneshvari Hospital, Shahid Beheshti University of Medical Sciences

## Abstract

The six major lineages of Mycobacterium tuberculosis [MTB] are found to be strongly associated with specific geographical outbreaks. But whether these bacterial lineages influence the host genetic polymorphism is uncertain. The present study was designed to evaluate the relevance of strain diversity and host genetic polymorphisms in susceptibility to pulmonary tuberculosis [PTB]. For this reason, single –nucleotide polymorphisms [SNPs] in interferon- γ [IFN-γ] receptor-1[G-611A], IFNG [G+ 2109A] and tumor necrosis factors [TNF-α] genes [at −238, 308,−857position] in patients [n=151] were analyzed and compared with controls [n=83]. The genetic diversity of M. tuberculosis isolates was performed using spacer oligonucleotide typing. Thereafter, the profile of IFN-γ and TNF-α allele frequency were investigated in each subtype of M.tuberculosis. The results showed C allele of TNF 857 and A allele of TNF 238 were more frequent in PTB cases [[TNF 857 C allele OR [CI95%] 0.6[0.4–0.9], p= 0.02] for TNF 238 A allele OR [CI95%] 5.5[3.4–9.0], p= 0.00]]. Similarly, G allele in IFNG+ 2109 A/G polymorphism were significantly more in patients than control subject[OR[CI95%] 0.3; p< 0.05]. The major identified clinical isolates of M. tuberculosis were EAI[42; 27.8% ], Haarlem[ 31; 20.5% ], CAS [ 23;15.2% ], Beijing[14; 9.2%], and T [11; 7.2% ] lineages. No correction was observed between strains diversity and frequency of SNPs in studied PTB cases. In conclusions, we exclude the possibility of genetic mutation in IFN-γ and TNF-α gene by different subtypes of M. tuberculosis. Although, our results supports a positive correlation between host SNPs and susceptibility to PTB.

## Introduction

Tuberculosis [TB], caused by *Mycobacterium tuberculosis*, is a major cause of morbidity and mortality throughout the world. It is estimated that one third of the world’s population is infected with *M. tuberculosis*, and approximately 1 billion people will be added to this number till 2020.^1^ Among those who are infected, only 5–10% will develop the active form of the disease with clinical symptoms. Other infected individuals may remain noninfectious and symptoms free for many years.^2^ Basically, the course of infection depends on a complex interaction of host, bacteria and environmental factors.^3^ The genetic contribution of the host in the individual susceptibility and development of disease is well studied during recent years.^3^ In this regards, both genes for interferon-gamma [IFN-γ] and tumor necrosis factor-alpha [TNF-α] have been identified as a essential components of the host immune response.^4^,^5^ IFN-γ is the key cytokine involved in the protective response against *M. tuberculosis* infection and is required for control of this pathogen. TNF-α in synergy with IFN-γ induce antimycobacterial activity of macrophages and increases its bactericidal activity.^6^ Till date, several polymorphisms within the promoter region of TNF-α and IFN-γ gene have been shown to be associated with susceptibility or resistance to TB in different ethnic groups.^7^,^8^,^9^,^10^ In contrast, the role of genetic variability of *M. tuberculosis* in the outcome of the infection remains to be uncertain.^11^ Most of immunological research on tuberculosis has been performed with laboratory strains i.e., H37RV and Erdman. But, with advances in molecular biology, it became apparent that *M. tuberculosis* is not a genetically conserved bacterium with limited phenotypic differences.^11^,^12^ Additionally, studies on molecular epidemiology showed differences in transmissibility and virulence of various subtypes of *M. tuberculosis*. Lopez and his co-workers were among the first investigators who could represent different immunopathological events using different *M. tuberculosis* strains.^11^ Later on, Tanveer *et al* studied the cytokine secretion in patients that were infected by CAS1 and Beijing subtypes of *M. tuberculosis*.^12^ In other studies, the influences of *M. tuberculosis* lineages to innate immune responses were characterized.^13^,^14^ However, association between host genetic polymorphisms and susceptibility to different lineages of *M. tuberculosis* strains was not reported. Recently, we showed the high prevalence of Beijing and Haarlem lineages among Iranian drug resistance TB patients.^7^,^15^ Initially, Beijing was described in 1995 as a closely related group of tubercle bacilli from the People’s Republic of China, and Haarlem were mainly found in Central America and Caribbean.^16^,^17^ To date, the prevalence of Beijing and Haarlem have reported in several countries.^16^,^18^ In the present study, the association of IFN-γ and TNF-α polymorphisms with susceptibility to TB in genetically diverse subtypes of *M. tuberculosis* are investigated. To our knowledge this is the first report that investigates association of host genetic polymorphism with genotyping of *M. tuberculosis*.

## Material and Methods

### Setting and study population

The study was conducted from January 2010 to December 2012, in the Mycobacteriology Research Center [MRC]. MRC is the only WHO-approved center for the detection and diagnosis of TB patients in Iran. A total of 151 patients with culture-positive TB and 83 healthy volunteers [referred to as normal controls] were included in the study. Patients and control subjects were matched for age, sex and nationality [The Institutional Review Board at the NRITLD approved the study and all the patients have signed informed consent].

### Mycobacterial isolates

Collected sputum samples from each patient were digested and decontaminated by Petroff’s method.^19^ Lowenstein-Jensen media were used for bacterial growth. The extracted DNA from culture positive samples was used for identification and spoligotyping.^15^,^20^ Drug susceptibility testing was performed against first –line anti TB drugs using proportional method.^21^

### Spoligotyping of MTB isolates

Spoligotyping was performed for all 151 clinical isolates according to the standard method.^20^ Briefly, DR region of mycobacterial genome was amplified by PCR using following primers: DRa 5′ - GGT TTT GGG TCT GAC GAC -3′ [biotinylated at 5′end] and DRb 5′-CCG AGA GGG GAC GGA AAC-3′ [Metabion, Martinsried, Germany]. The PCR amplicons were subsequently hybridized to a set of 43 different immobilized DR spacers covalently bound to the membrane. The hybridization signals were detected by chemiluminescence system [Amersham ECL detection kit, GE Healthcare Limited, UK] after incubation with a streptavidin-peroxidase conjugate [Roche, Germany]. DNA extracts of MTB H37Rv and *M.bovis* BCG were used as positive controls.

### Genetic evaluation

Genomic DNA was extracted using the standard protocol with slight modifications.^23^,^24^ Briefly, Peripheral Blood Leukocytes [PBLs] were separated from two milliliters of the whole blood using RBC lysis buffer [0.155 M NH_4_Cl, 0.01 M NaHCO_3_]. Thereafter, PBLs re-suspended in 500μl of SE buffer [NaCl 3M, EDTA 0.5M, PH=8], containing 40 μl of 10% SDS and 3μl of 20 mg/ml of proteinase K. The suspension was incubated at 60^0^C for 30 minutes. After incubation, 200μl of equilibrated phenol [PH=8] was added to the mixture and centrifuged for 10 min at 12000g. The aqueous phase transferred to a new tube and the DNA was precipitated using cold propanol.

### TNF-α genotyping

Polymorphisms in the TNF promoter region, namely TNF single nucleotide polymorphisms [SNP] 238, 308 and 857 were studied using PCR- RFLP. For TNF −308 polymorphisms, the following primers were used to amplify a 107bp product:5′ AGC AAT AGG TGG TTT TGA CTC GGGC CCAT-3′;5′TCC TCC CTG CTC CGA TTC CG-3′. For −238 polymorphisms, the following primers were used to amplify a 230 bp product : 5′CCT CAA GGA CTC CAA AGC TTT CTG -3′; 5′ACA CTC CCC ATC CTC CCA GATC -3′. For −857 polymorphisms, the following primers were used to amplify a 127 bp product: 5′ GGC TCT GAG GAA TGG GTT AC-3′ ;5′CCT CTA CAT GGC CCT GTC TAC-3′. The amplification was accomplished by an initial denaturation at 94°C for 5 min, and 30 cycles at 94°C for 40s, at 56°C for 40s, at 72°C for 1 min, followed by an extension at 72°C for 6 min [22,24]. PCR products of, TNF −238, TNF −308 and TNF −857 digested with 2 U enzymes of BgI II, Bsaj I, NcoI, TaiI and TaiI, respectively.^24^ Digested products were run on 8% polyacrylamide gel and were stained with Silver – Nitrate.

### IFN-γ genotyping

Single–nucleotide polymorphisms [SNPs] in interferon- γ [IFN-γ] receptor-1[G-611A], IFNG [G+ 2109A] were performed using PCR-RFLP. The target DNA was amplified in a PCR reaction mixture containing 1× reaction buffer [50 mM KCl, 67 mM Tris-HCl [pH 9.0], 2 mM MgCl2], 2 mM of dNTPs, 0.2U of *Taq* DNA-polymerase [Roch, Germany], and 20 pmol of each primers. For IFN-γ receptor-1[G-611A s, the following primers were used to amplify a 85bp product: 5′ AGACAAACCCAGAGAGGTAAGAGA3′; 5′ACCTTCTCAGCAATTCAGTGTCAAA3′. For IFNG [G+ 2109A] polymorphisms, the following primers were used to amplify a 366 bp Product; 5′AATCGCTGAAGTATGTAAT3′; 5′GCATTGTAGAGTTTTG3′. The PCR products of 78 [51.7%] were males; while in the control group 34 IFN-γ receptor-1[G-611A] and IFNG [G+ 2109A] digested with 2 U enzymes of NIaIII and FauI, respectively.^24^ Digested products were run on 8% polyacrylamide gel and were stained with Silver – Nitrate.

### Statistical analysis

Statistical analysis was performed using chi-square test to determine statistical associations between cases and control. P-value less than 0.05 were considered statistically significant. Data were analyzed using SPSS version 18 Software.

## Results

According to the demographic characteristics of studied populations, the mean age of patient and control groups was 48.7±22.1 and 32.7±6.4 years, respectively (Table 1). Majority of studied patient [47; 31.2%] had more than 65 years of old, followed by second group [42; 27.8 %] which had 25–44 years of ages. Seventy three [48.2%] TB cases were female and [42.2%] were females and 48 [57.8%] were males. Most of the patients [54.0%] were Iranian, and remains were immigrants.

### Spoligotyping

Of 151 MTB isolates for which spoligotyping was performed, 140 [92.6%] isolates were grouped into 13 different “shared type” that had been described in the SITVIT2 database and the remaining 11 [7.4%] isolates generated unknown spoligopatterns. The most frequent spoligotype in our populations belong to EAI [EAI1 and EAI3, n=42, 27.8%], Haarlem [H3 and H4, n=31, 20.5%] followed by CAS [CAS1 and CAS2, n=23, 15.2%], Beijing [n=14, 9.2%], and T [T, T3 and T4, n=11; 7.2%] lineages (Table 2).

### Drug susceptibility patterns

As shown in table 3, drug susceptibility testing of 151 strains indicated that 99 [65.6%] were sensitive to all tested agents and 52 [34.4%] were resistant to at least one drug. The majority of drug resistant isolates were resistance to INH [6.6%] followed by PZA [2.6%] and ETM [2.0%] and STM [2.0%]. None of the investigated isolated were RMP monoresistant. Twenty three isolates were MDR-TB [15.2%]. In an investigation between MTB strains and drug resistance we found that Beijing genotype was highly associated with MDR [p<0.05].

### Association of IFN-γ and TNF-α gene polymorphisms with TB

Allele and the genotype frequencies of investigated IFN-γ and TNF-α polymorphisms are enlisted in Table 4. In overall, three types of polymorphisms were observed in TNF-*α* gene: an A to G substitution at position −238, a G to A substitution at position −308, and a C to T substitution at position −857. Among these polymorphisms, C allele of TNF 857 and A allele of TNF 238 were more frequent in TB cases as compared to control group [TNF 857 C allele OR[CI95%] 0.6[0.4–0.9], p= 0.02] for TNF 238 A allele [OR[CI95%] 5.5[3.4–9.0], p= 0.00]. Additionally, TNF 857 C/C[ 85;56.2%] and TNF 238 A/A 127[84.1%] genotypes were associated with increased risk of acquiring TB. Two types of polymorphisms, in an A to G substitution at position + 2109 and −611, were observed for IFN-γ. The result showed in − 2109 A/G polymorphism, G allele were significantly more common in TB group [OR[CI95%] 0.3; p< 0.05]. The investigation of the allele and genotype frequencies for TNF- 308 and IFNR1 −611 polymorphisms revealed no significant association with resistance or susceptibility to TB [p > 0.05; Table 4].

### Association of IFN-γ and TNF-α polymorphisms with major lineages of M. tuberculosis

As shown in table 5, three polymorphic variants [two in TNF-*α* gene, and one in IFN-γ] are associated with susceptibility to TB. Distributions of these alleles are similar in patients that were infected with different subtypes of *M. tuberculosis*. For example, out of 42, 31, 23, 19, 14 TB patients infected with EAI, Haarlem, CAS, T, Beijing lineages, 88.6%, 87.1%, 91.2%, 86.4% and 87.5%, had TNF 238A allele, respectively. Similarly, the frequency of IFN-γ A allele was high in all TB patients [ranging from 61.1 to 86.4%]. Thereby, we found no correlation between host genetic polymorphisms and mycobacterial diversity.

## Discussion

Pathogenesis in tuberculosis is dependent on many components of the host, pathogen and environment.^27^ The present study was aimed to evaluate the possible correlation of host genetic polymorphisms with different genotypes of *M. tuberculosis*. Based on SIT from SITVIT2, the major identified clinical isolates *of M. tuberculosis* were EAI[42; 27.8%], Haarlem[ 31; 20.5%], CAS [ 23;15.2% ], Beijing [14; 9.2%], and T [11; 7.2%] lineages. Recently, it was shown that particular genotypes of *M. tuberculosis* could elicit different immune responses with high mortality rates in the course of experimental infection.^11^,^12^,^13^ For example, in mice model Beijing subtypes, induced early and massive pneumonia with death. Whereas, Canetti strains induced limited pneumonia with sustained expression of TNF-α.^27^,^29^ Likewise, other investigators showed a different level of cytokines production [TNF-α, IFN-γ] in patients infected with genetically distinct *M. tuberculosis* subtypes.^12^ Our results showed no association between the frequencies of SNPs in host and various lineages of *M. tuberculosis.* As shown in table 5, the TNF-α 238A and 857C alleles were associated with susceptibility to TB infection, but their distribution was almost equal among patient infected with different subtypes of *M. tuberculosis*. Likewise, the frequency of IFN2109A allele was high in TB patients than control subject, but no statistical differences were observed among the allele distribution in different *M. tuberculosis* lineages. Therefore, our results demonstrate no correlation between genetic diversity of *M. tuberculosis* and host susceptibility to TB. On the contrary to our results, Tanveer *et al* showed a correlation between cytokine induction i.e., TNF-α, IFN-γ and growth index of CAS1 and Beijing isolates in comparison to H37RV strain.^12^ Furthermore, they suggested that the phenotypic and genotypic polymorphisms in clinical *M. tuberculosis* strains may in turn influence the persistence and dissemination of differing genotypes.

At present, we have no explanation for such discrepancy, but we need more detailed studies in order to outline the importance of *M. tuberculosis* genotypes with host genetic polymorphism. Basically, genetic contribution of the host is an important factor in determining susceptibility to TB. Today, several cytokine gene polymorphisms have been described in association with susceptibility or resistance to TB. In present investigations, we found two polymorphisms of TNF gene promoter [−857 and −238] that were significantly associated with TB patients (Table 4). For TNF- 238 A/G SNP, A allele was more frequent in TB cases as compared to control. Previously also, positive association of TNF- 238 A/G was reported among Iranian pulmonary tuberculosis cases.^22^,^30^ TNF 238 A/G polymorphism has been extensively studied in TB cases of various ethnic groups.^8^,^9^,^10^ However, studies that were conducted in Turkey, India and Columbia demonstrated no association of specific allele of TNF-α gene with susceptibility to TB.^8^,^9^,^31^ There are also considerable variations in genotype frequencies of TNF 857 polymorphisms in different populations. TNF 857 T/C polymorphism in our study was significantly more frequent in TB cases as compared to control. However, conflicting reports are available about insignificant association of 857T/C genotype in Asian TB patients i.e. Indians.^4^ In fact, the contradictory data could be discussed in different ways; First of all, multiple polymorphisms within the TNF gene may have emerged during evolution in various ethnic groups to affect TB susceptibility or resistance. Second, the number of studied cases has a great impact on the outcome of the results. Generally, the large confidence intervals in some studies could be the result of the small sample size. For TNF 308 G/A, several studies on TB patients have produced approximately similar results. In recent surveys, no significant association in TNF 308 and TB were reported from Korea, Brazil and China, which is similar to our study.^9^,^32^,^33^

Another candidate gene for determination the susceptibility to TB is polymorphism in the IFN-γ gene.^3^,^26^ In different experimental set up, tuberculosis patients had deficient IFN-γ production in their peripheral blood mononuclear cells. Also, it has been shown that partial or complete loss of function alleles of IL-12/IFN-γ axis genes associated with diseases development.^13^,^14^,^34^ In the present study, IFN2109G allele was significantly associated with increased susceptibility to TB. However, we found no significant association between IFNR1611 A/G SNP and TB patients. Previously, Mirsaeidi *et al,* also did not find any significant association between IFNR1395 SNP and susceptibility to TB among Iranian studied cases.^35^ Also few studies declines the correlation of IFNGR1 polymorphism with *M. tuberculosis*, instead they proposed the correlation of IFNGR1 polymorphism with avirulent or *M bovis* BCG infection.^25^,^36^ These observations may outline the alternative pathways for enhancing host immune response against *M. tuberculosis*.

## Conclusions

Our findings showed that the polymorphisms in TNF-α promoter gene are likely associated with increased susceptibility to TB in Iranian patients. But, no significant association was found between frequencies of SNPs in host and genotyping of *M. tuberculosis*. However, further studies with multiple genes polymorphisms would be necessary to elucidate the exact role of *M. tuberculosis* genotyping.

## Figures and Tables

**Table 1:** Demographic of study populations

Characteristics	No. of cases [%]	

	TB patients	Control
Gender		
Female	73 [48.3]	35 [42.2]
Male	78 [51.7]	48 [57.8]
Age		
0–24	28 [18.5]	4 [4.8]
25–44	42 [27.8]	76 [91.6]
45–64	34 [22.5]	3 [3.6]
>65	47 [31.1]	0 [.0]
Nationality		
Iranian	82 [54.0]	83 [100.0]
Afghan	69 [46.0]	0 [0.0]
**Total**	**151 [100.0]**	**83 [100.0]**

**Table 2:** The spoligotyping pattern of MTB strains

SIT^a^	Family^b^	No. [%]^c^	Spoligopattern^d^
127	H4	27 [17.8]	■□■■■■■■■■■■■■■■■■■■■■■■■■■■□□□■□□□□■■■■■■■
337	EAI1	22 [14.5]	■■■■■■■■■■■■■■■■■■■■■■■■■■■□□□□□■□■■■■■□■■■
11	EAI3	20 [13.2]	■□□■■■■■■■■■■■■■■■■■■■■■■■■■□□□□■□■■□□□■■■■
26	CAS1	16 [10.6]	■■■□□□□■■■■■■■■■■■■■■■□□□□□□□□□□□□■■■■■■■■■
1	Beijing	14 [9.2]	□□□□□□□□□□□□□□□□□□□□□□□□□□□□□□□□□□■■■■■■■■■
54	Manu 2	9 [6.0]	■■■■■■■■■■■■■■■■■■■■■■■■■■■■■■■■□□■■■■■■■■■
53	T	8 [5.3]	■■■■■■■■■■■■■■■■■■■■■■■■■■■■■■■■□□□□■■■■■■■
357	CAS	7 [4.6]	■■■□□□□■■■■■■■■■■■■■■■□□□□□□□□□□□□□□■■■■■■■
602	U	7 [4.6]	■■■■■■■■■■■■■■■■■■■■■■■■□□□□□□□□□□□□■■■■■■■
294	H3	4 [2.6]	■□■■■■■■■■■■■■■■■■■■■■■■■■■■■■□■□□□□■■■■■■■
20	Lam1	3 [2.0]	■■□■■■■■■■■■■■■■■■■■□□□□■■■■■■■■□□□□■■■■■■■
37	T3	2 [1.3]	■■■■■■■■■■■■□■■■■■■■■■■■■■■■■■■■□□□□■■■■■■■
40	T4	1 [0.6]	■■■■■■■■■■■■■■■■■■□■■■■■■■■■■■■■□□□□■■■■■■■
Unknown	-	11 [7.4]	-
Total	-	151 [100.0]	-

a SIT from SITVIT2,

b Representing spoligotype families annotated in SITVIT2,

c Number of strains,

d [■] Presence of spacer; [□] absence of spacer

**Table 3:** Drug resistant patterns of MTB strains.

Type of resistance	No.[%] of strains									

	Haarlem	EAI	CAS	Beijing	T	MANU	U	LAM	Unknown	Total
Sensitivity to all drugs	19 [61.3]	28 [66.7]	19 [82.6]	4 [28.6]	8 [72.7]	6 [66.7]	4 [57.1]	2 [66.7]	9 [81.8]	99 [65.6]
MDR	2 [6.5]	5 [11.9]	1 [4.3]	10 [71.4]	1 [9.1]	2 [22.2]	1 [14.3]	1 [33.3]	0 [0.0]	23 [15.2]
INH resistance	2 [6.5]	4 [9.5]	1 [4.3]	0 [0.0]	1 [9.1]	0 [0.0]	1 [14.3]	0 [0.0]	1 [9.1]	10 [6.6]
STM resistance	1 [3.2]	1 [2.4]	0 [0.0]	0 [0.0]	0 [0.0]	1[11.1]	0 [0.0]	0 [0.0]	0 [0.0]	3 [2.0]
PZA resistance	4 [12.9]	0 [0.0]	0 [0.0]	0 [0.0]	0 [0.0]	0 [0.0]	0 [0.0]	0 [0.0]	0 [0.0]	4 [2.6]
ETM resistance	1 [3.2]	2 [4.8]	0 [0.0]	0 [0.0]	0 [0.0]	0 [0.0]	0 [0.0]	0 [0.0]	0 [0.0]	3 [2.0]
INH/STM resistance	0 [0.0]	1 [2.4]	1 [4.3]	0 [0.0]	1 [9.1]	0 [0.0]	1 [14.3]	0 [0.0]	0 [0.0]	4 [2.6]
RMP/STM resistance	2 [6.5]	1 [2.4]	1 [4.3]	0 [0.0]	0 [0.0]	0 [0.0]	0 [0.0]	0 [0.0]	1 [9.1]	5 [3.3]
Total	31 [100.0]	42 [100.0]	23 [100.0]	14 [100.0]	11 [100.0]	9 [100.0]	7 [100.0]	3 [100.0]	11 [100.0]	151 [100.0]

**Table 4:** Allele and genotype frequencies in TB cases

	Control [83]	TB cases [151]	OR [CI 95%]	P
**TNF857**				
Allele				
T	56 [33.7]	72 [23.8]	0.6 [0.4–0.9]	0.02
C	110 [66.3]	230 [76.2]		
Genotype				
TT	8 [9.6]	6 [3.9]	0.3 [0.4–1.1]	0.08
TC	40 [48.1]	60 [39.7]	0.7 [0.4–1.2]	0.2
CC	35 [42.1]	85 [56.2]	0.5 [0.3–0.9]	0.03
**TNF308**				
Allele				
G	154 [92.7]	272 [90.0]	0.7 [0.3–1.4]	0.3
A	12 [7.3]	30 [10.0]		
Genotype				
GG	71 [85.5]	122 [80.7]	0.7 [0.3–1.4]	0.3
GA	12 [14.5]	28 [18.5]	1.3 [0.6–2.8]	0.4
AA	0 [0.0]	1 [0.6]	1.5 [1.4–1.7]	0.4
**TNF238**				
Allele				
A	100 [60.2]	270 [89.4]	5.5 [3.4–9.0]	0.00
G	66 [39.8]	32 [10.6]		
Genotype				
AA	49 [59.0]	127 [84.1]	3.6 [1.9–6.8]	0.00
AG	2 [2.4]	16 [10.5]	4.8 [1.0–21.4]	0.02
GG	32 [38.5]	8 [5.2]	0.1 [0.03–0.2]	0.00
**IFNR1611**				
Allele				
A	118 [71.0]	237 [78.4]	1.4 [0.9–2.2]	0.07
G	48 [29.0]	65 [21.6]		
Genotype				
AA	47 [56.6]	97 [64.2]	1.3 [0.7–2.3]	0.2
AG	24 [28.9]	43 [28.4]	0.9 [0.5–1.7]	0.9
GG	12 [14.4]	11 [7.2]	0.4 [0.1–1.1]	0.07
**IFN2109**				
Allele				
A	152 [91.5]	241 [79.8]	0.3 [0.1–0.6]	0.001
G	14 [8.5]	61 [20.2]		
Genotype				
AA	69 [83.1]	97 [64.2]	0.3 [0.1–0.7]	0.002
AG	14 [16.9]	47 [31.1]	2.2 [1.1–4.3]	0.01
GG	0 [0.0]	7 [6.4]	1.5 [1.4–1.7]	0.04

**Table 5:** Allele and genotype frequencies in different genotypes of MTB

	TB cases [151]	Haarlem [31]	P	Beijing [14]	P	CAS [23]	P	EAI [42]	P	T [11]	P	MANU [9]	P	U [7]	P	LAM [3]	P
**TNF857**																	
Allele																	
T	72 [23.8]	15 [24.2]	0.9	10 [35.7]	0.1	13 [28.9]	0.4	18 [21.4]	0.6	5 [22.7]	0.9	4 [22.2]	0.8	5 [35.7]	0.3	0 [0.0]	0.3
C	230 [76.2]	47 [75.8]		18 [64.3]		33 [71.1]		66 [78.6]		17 [77.3]		14 [77.8]		9 [64.3]		3 [100.0]	
Genotype																	
TT	6 [3.9]	2 [6.5]	0.5	1 [7.1]	0.5	1 [4.3]	0.9	1 [2.4]	0.6	1 [9.1]	0.4	0 [0.0]	0.5	1 [14.3]	0.1	0 [0.0]	0.7
TC	60 [39.7]	11 [35.5]	0.6	8 [57.1]	0.2	11 [47.8]	0.4	16 [38.1]	0.8	3 [27.3]	0.4	4 [44.4]	0.7	3 [42.9]	0.8	0 [0.0]	0.1
CC	85 [56.3]	18 [58.1]	0.8	5 [35.5]	0.1	11 [47.8]	0.4	25 [59.5]	0.7	7 [63.6]	0.6	5 [55.6]	0.9	3 [42.9]	0.4	3 [100.0]	0.1
**TNF238**																	
Allele																	
A	270 [89.4]	54 [87.1]	0.5	26 [87.5]	0.5	42 [91.2]	0.6	76 [88.6]	0.7	19 [86.4]	0.6	15 [83.3]	0.4	14 [100.0]	0.1	5 [83.3]	0.6
G	32 [10.6]	8 [12.9]		2 [12.5]		4 [8.8]		8 [11.4]		3 [13.6]		3 [16.7]		0 [0.0]		1 [16.7]	
Genotype																	
AA	127 [84.1]	26 [83.9]	0.9	13 [92.9]	0.3	19 [82.6]	0.8	36 [84.5]	0.7	9 [81.8]	0.8	6 [66.7]	0.1	7 [100.0]	0.2	2 [66.7]	0.4
AG	16 [10.5]	2 [6.5]	0.4	0 [0.0]	0.2	4 [17.4	0.3	4 [10.4]	0.8	1 [9.1]	0.8	3 [33.3]	0.04	0 [0.0]	0.3	1 [33.3]	0.2
GG	8 [5.2]	3 [9.7]	0.3	1 [7.1]	0.7	0 [0.0]	0.2	2 [4.8]	0.8	1 [9.1]	0.5	0 [0.0]	0.4	0 [0.0]	0.5	0 [0.0]	0.6
**IFN2109**																	
Allele																	
A	241 [79.8]	47 [75.8]	0.4	20 [68.7]	0.2	38 [82.6]	0.6	71 [84.5]	0.3	19 [86.4]	0.4	11 [61.1]	0.06	12 [85.7]	0.5	5 [83.3]	0.8
G	61 [20.2]	15 [24.2]		8 [31.3]		8 [17.4]		13 [15.5]		3 [13.6]		7 [38.9]		2 [14.3]		1 [16.7]	
Genotype																	
AA	97 [64.2]	17 [54.8]	0.3	7 [50.0]	0.2	16 [69.6]	0.6	31 [73.8]	0.2	8 [72.7]	0.5	4 [44.4]	0.2	5 [71.4]	0.6	2 [66.7]	0.9
AG	47 [31.1]	13 [41.9]	0.2	6 [42.9]	0.3	6 [26.1]	0.6	9 [21.4]	0.2	3 [27.3]	0.7	3 [33.3]	0.8	2 [28.6]	0.8	1 [33.3]	0.9
GG	7 [4.6]	1 [3.2]	0.7	1 [7.1]	0.6	1 [4.3]	0.9	2 [4.8]	0.9	0 [0.0]	0.4	2 [22.2]	0.02	0 [0.0]	0.5	0 [0.0]	0.7
